# Resistance mechanisms and prospects of trastuzumab

**DOI:** 10.3389/fonc.2024.1389390

**Published:** 2024-11-25

**Authors:** Lizhe Wang, Yu Wang, Yueyang Li, Li Zhou, Jiahui Du, Jin Wang, SiHan Liu, Yongyi Cao, Yuzhi Li, Wenying Yang, Ting Zhu

**Affiliations:** The Third Affiliated Hospital of Anhui Medical University, Hefei first people’s Hospital, Hefei, China

**Keywords:** trastuzumab, HER2, breast cancer, tumor microenvironment, resistance

## Abstract

Breast cancer that overexpresses Human Epidermal Growth Factor Receptor 2 (HER2+) due to gene amplification or overexpression constitutes 15-20% of all breast cancer cases. Trastuzumab, the first FDA-approved monoclonal antibody targeting HER2, serves as the standard first-line treatment for HER2-positive advanced breast cancer, as recommended by multiple clinical guidelines.Currently, accumulated clinical evidence reveals a considerable degree of variability in the response of HER2+ breast cancer to trastuzumab treatment. Specifically, over 50% of patients either do not respond to or develop resistance against trastuzumab.The specific mechanisms of resistance to trastuzumab are currently unclear. This paper aims to review the existing research on the resistance mechanisms of trastuzumab, based on its target, from aspects such as genetic loci, molecular structure, signaling pathways, and the tumor microenvironment and to outline current research progress and new strategies.

## 1 Introduction

The prevalence of breast cancer (BC) among women has risen to become the leading cause of cancer incidence globally, with a considerable associated mortality rate. Among the molecular subtypes, HER2-positive breast cancer accounts for 15-20% of cases and is often associated with high invasiveness, metastatic potential, and treatment resistance, serving as a predictor of poor prognosis (1). Since its approval by the FDA in 1998, trastuzumab has emerged as a groundbreaking anti-HER2 monoclonal antibody. By binding to the extracellular domain (ECD) of HER2, trastuzumab effectively blocks intracellular HER2 signaling pathways, leading to cell cycle arrest and antibody-dependent cellular cytotoxicity. These mechanisms have collectively contributed to significant improvements in the prognosis of HER2-positive patients (2). However, a substantial body of clinical and research evidence indicates that resistance to trastuzumab is inevitable. The mechanisms of trastuzumab resistance remain unclear, with prevailing hypotheses including HER2 mutations or isoform formation, activation of alternative signaling pathways, epitope masking, and changes in the tumor microenvironment, among others. These challenges have led to the development of new treatment strategies, such as antibody-drug conjugates and dual antibody therapies. This article aims to provide a comprehensive overview of the resistance mechanisms of trastuzumab through its interaction with HER2 (3, 4).

## 2 HER family

The members of the HER family, HER1 (EGFR, ERBB1), HER2, HER3 (ERBB3), and HER4 (ERBB4), play a central role in regulating cell proliferation and differentiation in embryonic and human tissues (5). They are critical in the upregulation, mutation, and hyperactivation of human cancers due to their structural similarities and specificity (6). These receptors use multiple activation mechanisms to identify growth factors through diverse recognition methods. They are crucial in regulating cancer phenotypes by interacting with proximal signal transduction components, thereby serving as primary targets for drugs designed to treat tumor cells (7).

## 3 HER2

The HER2 gene, also known as Neu, ErbB2, or CD340, was initially identified in human breast cancer cells (8). It belongs to the receptor tyrosine kinase (RTK) family, specifically the epidermal growth factor receptor (EGFR) subfamily. HER2 is a tyrosine kinase receptor membrane glycoprotein encoded by the ErbB gene, located on the long arm of chromosome 17 in band 2 region 1 (17q21), and is classified as an oncogene (9). The protein encoded by this gene undergoes glycosylation, has a molecular weight of 185 kDa, and is anchored to the cell membrane. This transmembrane protein consists of three distinct domains: the extracellular domain (ECD), the transmembrane domain (TM), and the intracellular domain (ICD). These domains work together to form the complete structure of the transmembrane protein (10).

## 4 HER Receptor Activation

The activation of HER family members primarily depends on autocrine or paracrine pathways (7).

ErbB receptors feature an extracellular region of about 630 amino acids, structured into four unique domains: I/L1, II/CR1, III/L2, and IV/CR2. These domains are sequentially organized, alternating in pairs. The receptors also include a single transmembrane domain and a cytoplasmic tyrosine kinase (9). Each structural domain includes specific sub-domains and amino acid residues, which can be classified into ligand-binding and dimerization domains. Except for HER3, all receptors contain a cytoplasmic tyrosine kinase region. Except for HER2, all receptors bind specific ligands through their extracellular domains. Upon dimerization, HER2 exhibits strong kinase activity and preferential binding. The dimer features leucine-rich residues in extracellular sub-domains I and III and cysteine residues in sub-domains II and IV, primarily connected by disulfide bonds (10, 11).

Tyrosine kinase phosphorylation is triggered through heterodimerization, leading to the activation of several signaling pathways, notably Ras/MAPK, PI3K/AKT/mTOR, PLCγ/PKC, and JAK2/STAT. These pathways play essential roles in key biological processes, including cell growth, programmed cell death, differentiation, blood vessel formation, and cellular invasiveness (12, 13).

## 5 Mechanisms of trastuzumab resistance

### 5.1 Gene overexpression or deletion

After anti-HER2 treatment, the overexpression or deletion of associated genes can result in mutations in the target itself, causing decreased or lost HER2 expression, which in turn diminishes drug efficacy. Research on resistance to trastuzumab has identified mutations in genes such as ATM, CDH1, GNAS, MLH1, RB1, SMARCB1, SMO, TP53, and VHL (14). The p53 signaling pathway is activated by DNA damage, emphasizing its vital role in cellular stress response mechanisms (15). These mutations may contribute to the development of resistance to therapy (16).

For example, ATM and RB 1 are mainly associated with cell cycle regulation and cellular senescence, and have significant associations with drug resistance in trastuzumab. Studies have shown that ATM (ataxia telangiectasia mutated) plays a key role in the DNA damage response, and that cells lacking ATM show resistance to apoptosis induced by low levels of DNA double-strand breaks, which may lead to early senescence1 (17). Moreover, RB 1 (retinoblastoma protein), as an important tumor suppressor, regulates the cell transition from G1 to S phase, and its loss of function is closely related to the development of various cancers. In HER 2-positive breast cancers, trastuzumab resistance is often associated with dysregulation of cell cycle regulation, especially as inactivation of RB 1 may lead to cellular resistance to therapy (18).

The interaction between ATM and RB1 may significantly influence the regulation of cell proliferation and apoptosis. ATM modulates cell proliferation and the apoptotic response by controlling the levels of the ARF (alternative reading frame) tumor suppressor protein. In contrast, RB1 regulates cell cycle progression by interacting with E2F transcription factors, thereby impacting cell growth and treatment response. Consequently, the dysregulation of ATM and RB1 might be a crucial factor underlying the resistance of HER2-positive breast cancer cells to trastuzumab. The role of ATM and RB1 in cell cycle control and cellular senescence, as well as their connection to trastuzumab resistance, offers important insights into the mechanisms underlying therapeutic resistance in breast cancer (19).

### 5.2 HER2 density or structural changes

#### 5.2.1 HER2 mutations or isoform formation

Due to the widespread presence of mutations, splicing, and post-translational modifications in tumor cells, HER2 inevitably produces isoforms and various subtypes. Current research indicates the existence of HER2 splicing variants lacking exon 16 (HER2Δ16), and the co-expression of HER2 subtypes has a unique impact on the phenotype of breast tumors. The activation of oncogenes, with a primary mechanism closely tied to epithelial-mesenchymal transition (EMT), is integral to this process (20). The formation of stable disulfide bonds at the molecular level eventually activates downstream signaling pathways, potentially causing a delayed response and reduced efficacy of trastuzumab (21).

**Figure 1:**
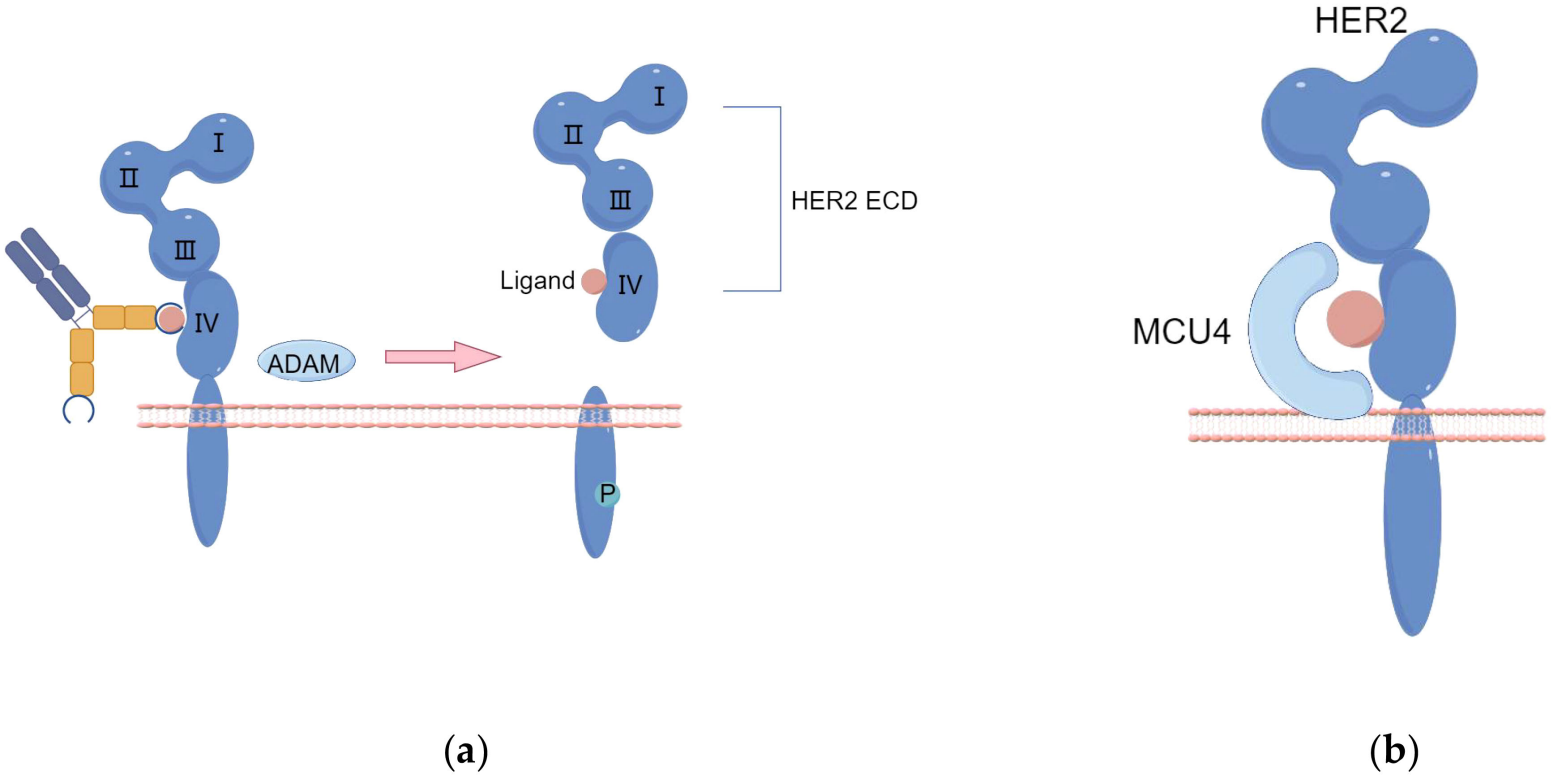
**(A)** The proteolytic cleavage of HER2-ECD by ADAM enzymes results in the production of a truncated form of HER2 protein, referred to as p95HER2. **(B)** Enhanced activation of the Signal Transducer and Activator of STAT3 pathway leads to the upregulation of MUC1/MCU4 expression, subsequently contributing to the development of resistance against trastuzumab. (By Figdraw).

Under certain conditions, proteolytic cleavage can result in the formation of specific mutant forms of the epidermal growth factor receptor. For example, soluble EGFR (sEGFR) can be produced either by selective splicing of receptor mRNA or by site-specific proteolytic cleavage of the receptor, releasing the extracellular domain. Similar to EGFR, matrix metalloproteinase (MMP) and ADAM (A Disintegrin And Metalloprotease domain) cleavage of HER2-ECD produces a truncated form of HER2, known as p95HER2—a 95 kDa truncated fragment of the HER2/neu protein. The absence of the extracellular domain in p95HER2 leads to resistance to trastuzumab, and p95HER2 can form homodimers, bypassing the binding site of trastuzumab or diminishing its effect, thereby activating multiple downstream pathways (**Figure 1A**). This variant is prevalent in cancer patients, accounting for 22.4%, and has been closely associated with breast cancer metastasis and recurrence (22, 23).

Moreover, the truncated form of the extracellular domain of the antigen might represent another possible resistance mechanism, although this has not yet been clinically confirmed for trastuzumab deruxtecan. This concept has, however, been previously demonstrated with trastuzumab; tumors expressing the complete receptor responded well to trastuzumab, whereas those expressing the truncated p95HER2 form showed resistance (24).

#### 5.2.3 HER2 epitope masking

Trastuzumab resistance primarily arises from antibody binding obstruction, which is facilitated by MUC4. MUC4 covers the trastuzumab binding site on HER2, leading to trastuzumab resistance upon prolonged exposure (**Figure 1B**) (25). TNFα-induced expression of MUC4 leads to trastuzumab resistance, impairing Antibody-Dependent Cellular Cytotoxicity (ADCC). CD44, a transmembrane hyaluronic acid receptor overexpressed in JIMT-1 cells, is proven to interact with polymerized hyaluronic acid.

**Figure 2:**
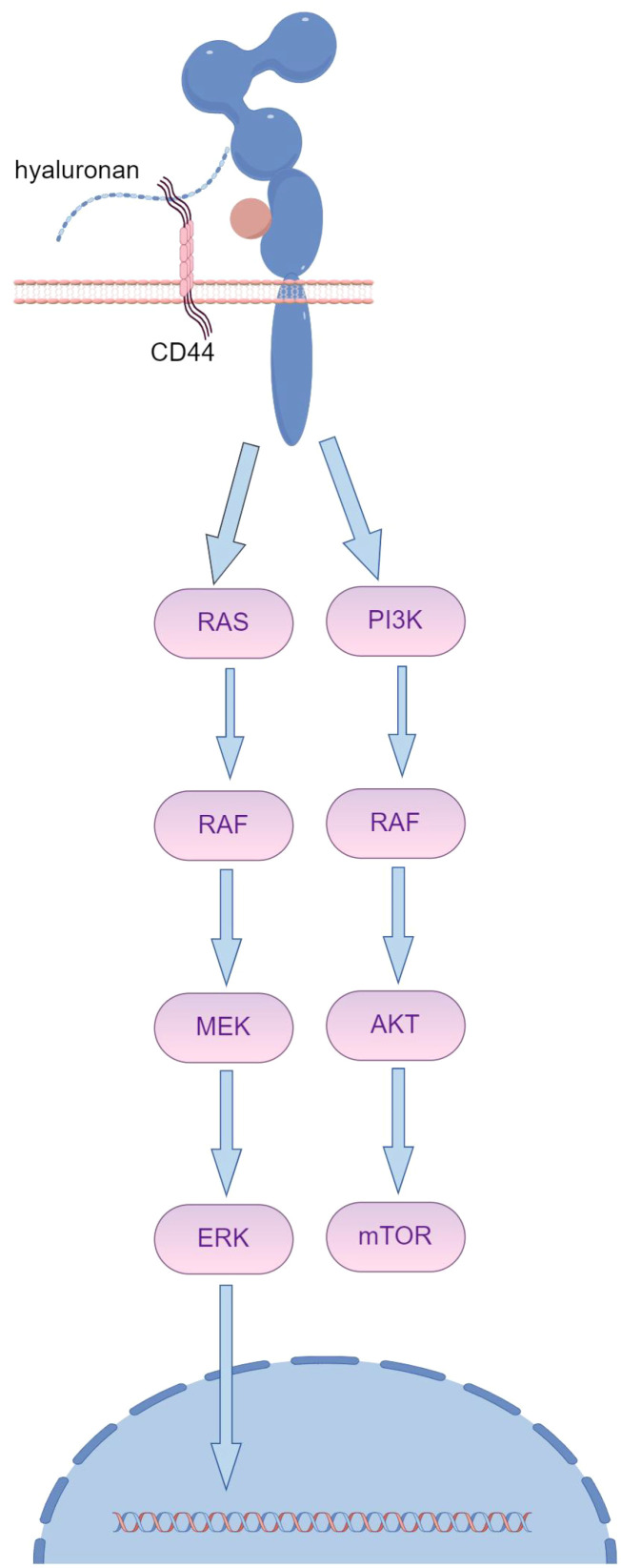
The overexpression of ECM components, such as MUC4 or CD44/hyaluronic acid, creates a spatial barrier that shields the HER2 antigen from recognition, subsequently leading to resistance to trastuzumab. (By Figdraw).

Although HER2-targeted antibodies have been effective for treating HER2-positive cancers, a considerable number of patients do not respond despite sustained antigen expression. Overexpression of extracellular matrix (ECM) components, such as MUC4 or CD44/hyaluronic acid, creates a physical barrier that masks the HER2 antigen, hindering its recognition (**Figure 2**). Multichannel targeted delivery approaches, like the synergistic action of KLA-R16, survivin siRNA, and Herceptin, can help mitigate resistance in recurrent breast cancer driven by CD44 overexpression (26).

In preclinical models, HER2-redirected CAR T cells have shown considerable potential in eliminating HER2-positive trastuzumab-sensitive tumor cell lines, such as SKBR3 and BT474. Moreover, clinical trials have demonstrated that these CAR T cells can effectively treat trastuzumab-resistant patients. However, evidence of their anti-tumor efficacy in trastuzumab-resistant tumors is still lacking (27). ECM components contribute to trastuzumab resistance by sterically hindering antigen recognition, which could also impact the effectiveness of emerging antibody-drug conjugates (ADCs). Therefore, the clinical application of trastuzumab-derived HER2-specific CAR T cells may provide a promising option for treating trastuzumab-resistant tumors (28).

#### 5.2.4 HER2 shedding

Recent studies suggest that dysregulation in the shedding of extracellular domains (ECD) from transmembrane receptors may contribute to tumor progression and resistance. Specifically, the shedding of the HER2 receptor results in the release of soluble HER2-ECD, which retains the trastuzumab recognition epitope while leaving behind the oncogenic, membrane-associated p95HER2 fragment. The presence of p95HER2 plays a significant role in trastuzumab resistance (29).

Studies have found that upregulating DPAGT1 persistently promotes HER2 shedding, leading to resistance to trastuzumab (30).

#### 5.2.5 Heterodimer formation

**Figure 3:**
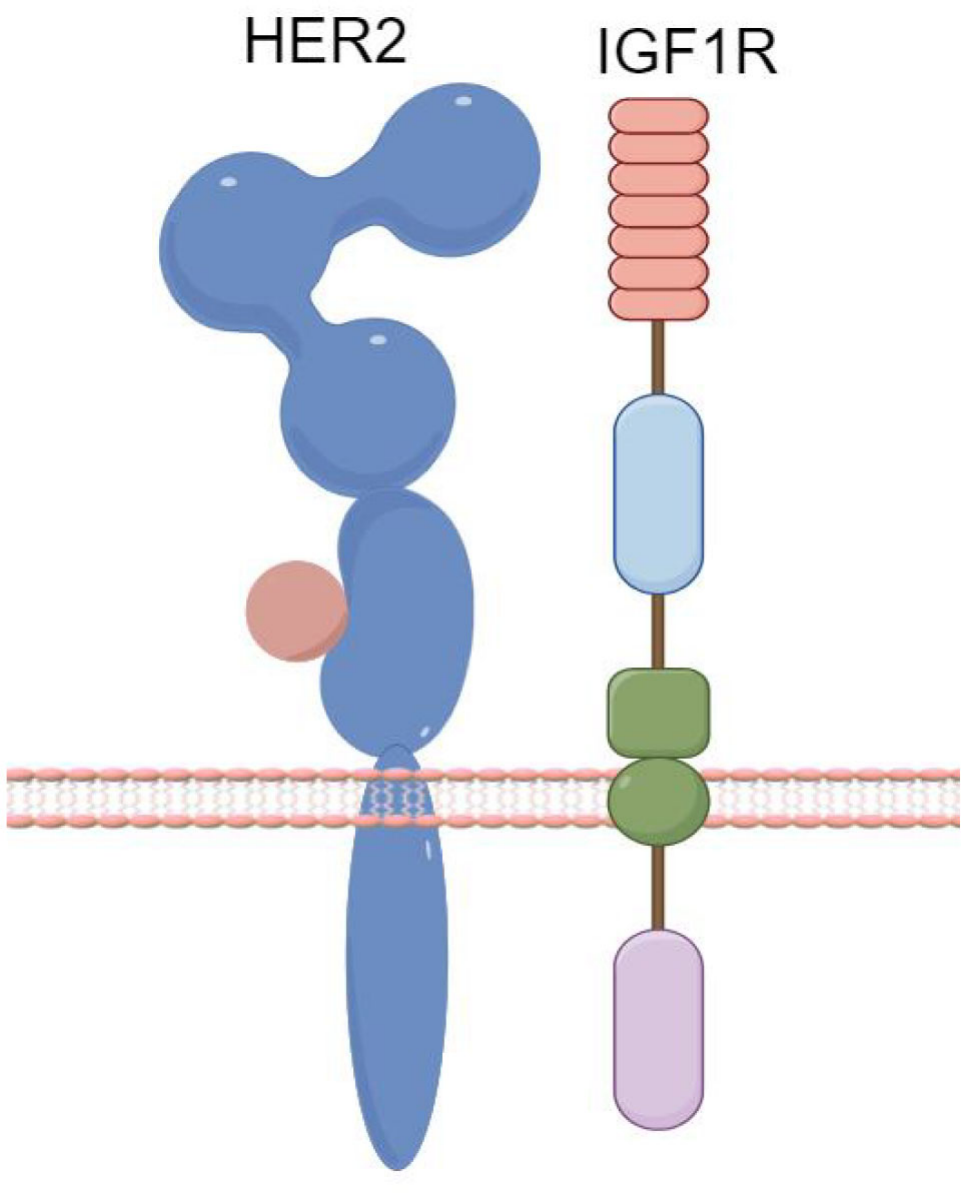
Formation of IGF1R/HER2 Heterodimers. (By Figdraw).

Recent research indicates that increased IGF2 protein expression is observed in trastuzumab-resistant breast cancer strains, which may be linked to the formation of IGF1R/HER2 heterodimers (**Figure 3**). It has been demonstrated that miR-98-5p can bind to the 3’UTR of IGF2 mRNA (31). Additionally, a significant correlation has been identified between the downregulation of miR-98-5p, the upregulation of IGF2, and trastuzumab responsiveness. These factors may thus serve as predictive markers for trastuzumab sensitivity and as potential targets to combat trastuzumab resistance.

AXL, a member of the TAM (TYRO3, AXL, MER) receptor tyrosine kinase family, is associated with epithelial-mesenchymal transition. The formation of HER2 heterodimers has been linked to trastuzumab resistance (32). AXL activates the PI3K/AKT and MAPK pathways through binding with its ligand, Growth Arrest-Specific 6 (GAS6), extracellular domain-mediated dimerization, or through signaling cascade formation with other transmembrane receptors, ultimately leading to resistance phenotypes. Combined targeting of AXL and anti-HER2 therapies could be a potential strategy to overcome trastuzumab resistance (33).

## 6 Activation of the downstream signaling pathway of HER2

### 6.1 Constitutive activation of the PI3K/AKT pathway

**Figure 4:**
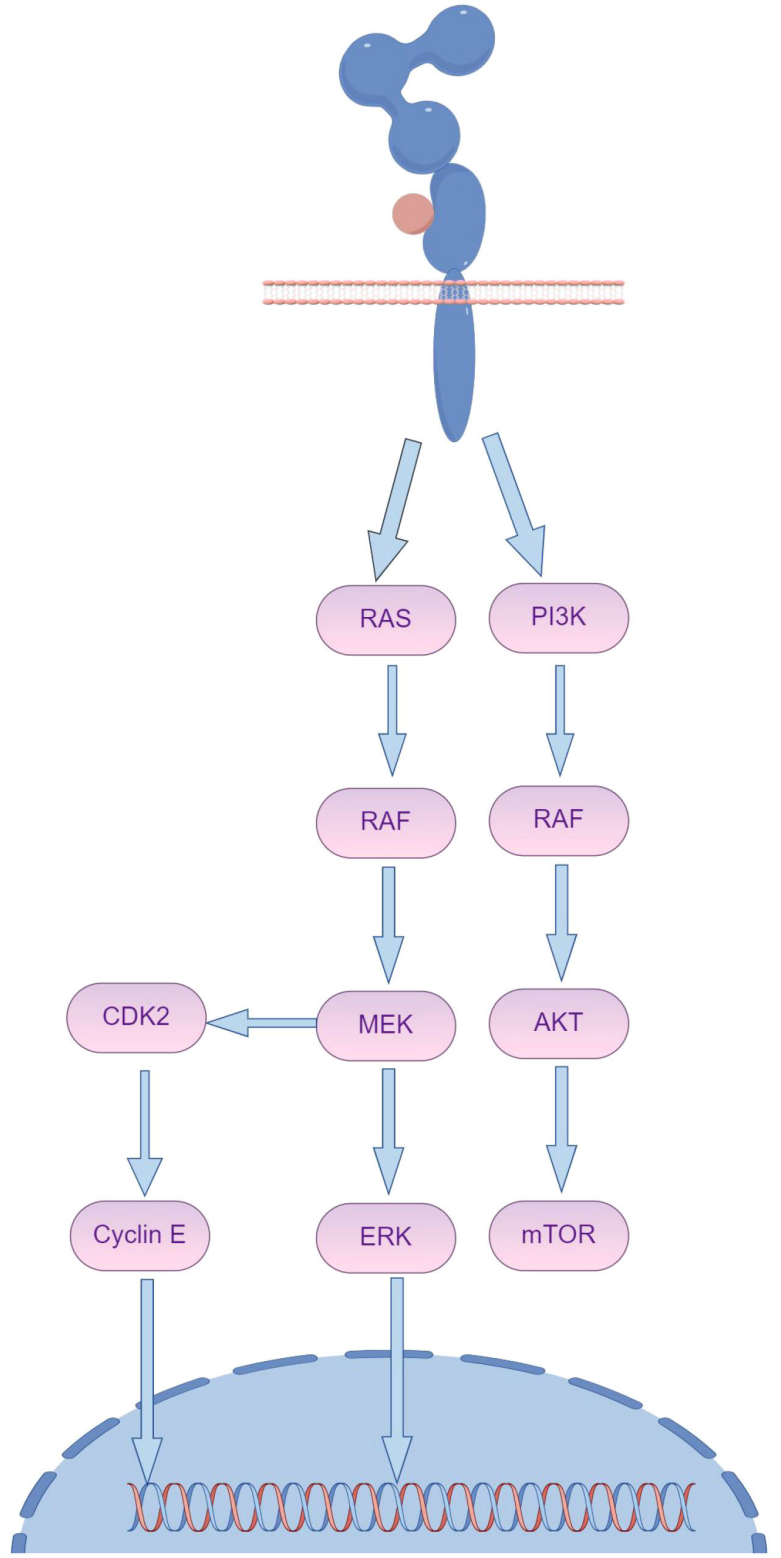
The activation of HER2 downstream signaling pathways, notably the persistent activation of PI3K/AKT and collateral loops, plays a crucial role in maintaining cellular function and survival. (By Figdraw).

Recent research highlights that the primary downstream signaling pathways of HER2 are RAS/RAF/MEK/ERK and PI3K/AKT, with mutations in the PI3K/AKT pathway accounting for a significant portion of resistance cases (**Figure 4**) (34). Specifically, mutations in PI3K prevent the inhibition of AKT even when the complex is disrupted, allowing downstream signaling to negate trastuzumab’s inhibitory effects on HER2 (35). The use of multiple targeted therapies has shown effectiveness in addressing this resistance. The HER family is essential for tumor cell proliferation, regulated by various ligands. Upregulation of integrins has been closely associated with trastuzumab resistance, particularly due to the activation of the PI3K pathway. Targeting both HER2 and integrins can partially mitigate trastuzumab resistance driven by PI3K activation. Recombinant protein-drug conjugates, RP-HI and RPDC-HI, have been developed to target these interactions, blocking the HER family-integrin interactions and subsequently inhibiting downstream signaling, thus alleviating trastuzumab resistance (36).

**Figure 5:**
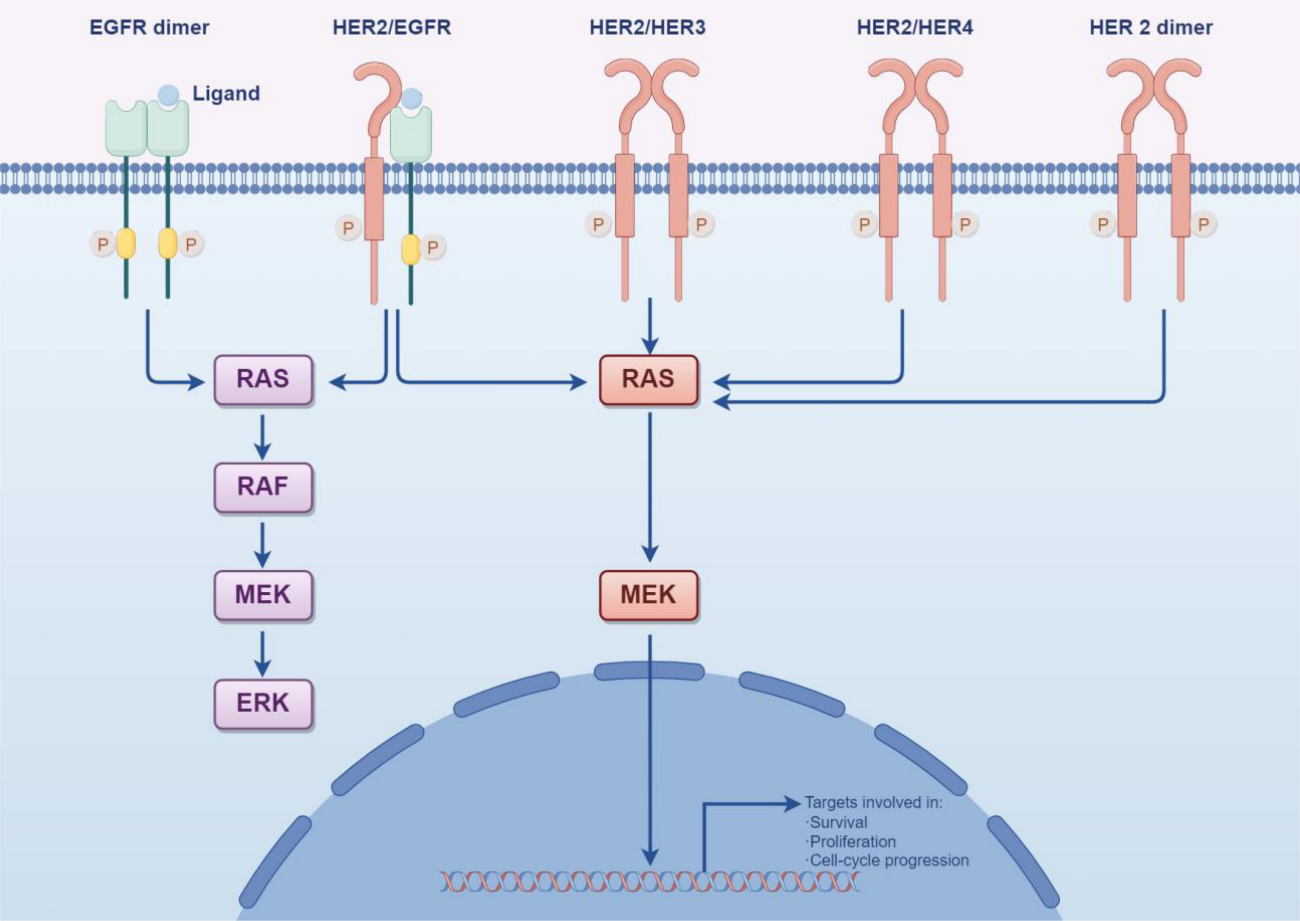
HER2 can create a “homodimer” as well as form “heterodimers” with HER1, HER3, and HER4. These various dimers significantly activate the PI3K/Akt and MAPK signaling pathways within cells, thereby controlling processes such as tumor cell proliferation, differentiation, migration, and apoptosis. This activation ultimately reduces the effectiveness of trastuzumab and contributes to drug resistance over time. (By Figdraw).

Activation of certain signaling pathways may contribute significantly to resistance in malignant cells against antibody-drug conjugates (ADCs). One such pathway is PI3K/AKT/mTOR, which plays a key role in cell survival, growth, and metabolic processes (**Figure 5**). Activation of this pathway has been linked to decreased ADC sensitivity, which reduces the efficacy of the cytotoxic payloads carried by ADCs and prolongs cell survival (31, 37). This phenomenon has been observed in patients carrying PIK3CA mutations or PTEN deletions during trastuzumab treatment. Specifically, PTEN downregulation has been linked to trastuzumab treatment failure, suggesting that PTEN deficiency or PIK3CA hyperactivation reduces trastuzumab sensitivity by activating the PI3K/AKT pathway. These findings highlight the importance of considering inhibitors targeting these signaling pathways in future therapeutic approaches to improve the effectiveness of trastuzumab and related drugs and overcome resistance issues.

The cyclin-dependent kinase inhibitor protein p27Kip1 is a critical factor in trastuzumab responsiveness, and its downregulation has been shown to promote trastuzumab resistance. Additionally, activation of the cyclin D1-CDK4/6 (cyclin-dependent kinase 4/6) pathway has been found to limit the antitumor activity of HER2 inhibitors *in vivo*. The combined use of CDK4/6 inhibitors with anti-HER2 and endocrine therapies has shown promising potential in treatment strategies, providing a potential avenue for overcoming trastuzumab resistance (37).

### 6.2 PTEN loss

The loss of PTEN leads to the continuous activation of the PI3K/AKT signaling pathway. In HER2-positive cancers, decreased PTEN expression or PTEN polymorphism hinders trastuzumab-mediated growth inhibition (38). PTEN levels vary between HER2-negative and HER2-positive cancers (39).

## 7 Tumor microenvironment

### 7.1 Immune-mediated resistance

#### 7.1.1 ADCC escape

ADCC, standing for Antibody-Dependent Cellular Cytotoxicity, is an immune response capable of lysing target cells. The immune system possesses a crucial defense mechanism, a nonspecific cytotoxic reaction that involves a range of immune cells, namely natural killer cells, macrophages, and monocytes (40).

#### 7.1.2 ADCC regulation

Human Leukocyte Antigen G (HLA-G) is a non-classical MHC I molecule. In the tumor microenvironment, trastuzumab induces abnormal production of TGF-β and IFN-γ, which subsequently enhances the HLA-G/KIR2DL4 signaling pathway, where HLA-G is the sole ligand for KIR2DL4 (41). In breast cancer cells that exhibit high HLA-G expression, binding to KIR2DL4 on natural killer (NK) cells suppresses their anti-tumor activity, thereby reducing the therapeutic efficacy of trastuzumab. Experimental studies suggest that disrupting the HLA-G/KIR2DL4 pathway can enhance the immune response of NK cells against breast cancer cells, thereby improving trastuzumab’s anti-tumor effect (42).

Given the significant role of antibody-dependent cellular cytotoxicity (ADCC) in trastuzumab’s immune response against cancer, a more strategic combination therapy approach could be beneficial for HER2-positive cancer (43). However, IFN-γ also induces PD-L1 expression in HER2-overexpressing breast cancer cells. Altogether, these findings highlight the potential of combining HLA-G and PD-L1/PD-1 targeted therapies as a strategy to address trastuzumab resistance (44, 45).

### 7.2 Adaptive immunity

Recent studies have shown that during treatment with trastuzumab, it can induce human peripheral blood mononuclear cells (PBMCs) or natural killer cells (NK cells) to secrete interferon-gamma (IFN-γ) (46). This secretion of interferon-gamma (IFN-γ) can prompt cancer cells to increase the expression levels of Major Histocompatibility Complex-I (MHC-I) and CD86.Ultimately, this upregulated expression of MHC-I and CD86 can enhance T-cell-mediated anti-tumor immune responses (47).

The Fc gamma receptor (FcγR) plays a crucial role in the anti-tumor action of trastuzumab. Traditionally, FcγR was thought to be expressed only in cells of the hematopoietic system, but recent studies have shown its presence in non-leukocyte cells, such as fibroblasts. In breast cancer, high infiltration of CD16+ fibroblasts correlates with poor prognosis and trastuzumab resistance (48). The Rho family guanine nucleotide exchange factor VAV2 is essential for the function of CD16 in fibroblasts, and targeting VAV2 can potentially reverse trastuzumab resistance. Recent research involving HER2-positive breast cancer patients and experimental models also suggests that the gut microbiome may act as an exogenous tumor factor contributing to cancer progression and treatment resistance (49).

Cancer-associated fibroblasts (CAFs) constitute a significant population of cells within the tumor microenvironment (TME) across various cancer types, breast cancer included (50). Research indicates that immunosuppressive cells resident in the TME, specifically CAFs and cancer-associated macrophages, play a role in immune evasion by tumor cells (51). In the context of cancer, CAFs have been shown to facilitate the migration of monocytes, leading to their transition towards the M2 phenotype through the secretion of factors such as chitinase 3-like 1 (Chi3L1). Nevertheless, the precise mechanism by which CAFs contribute to tumor resistance against trastuzumab-mediated ADCC remains elusive and warrants further investigation (52, 53).

## 8 The association between angiogenesis and trastuzumab resistance

**Figure 6:**
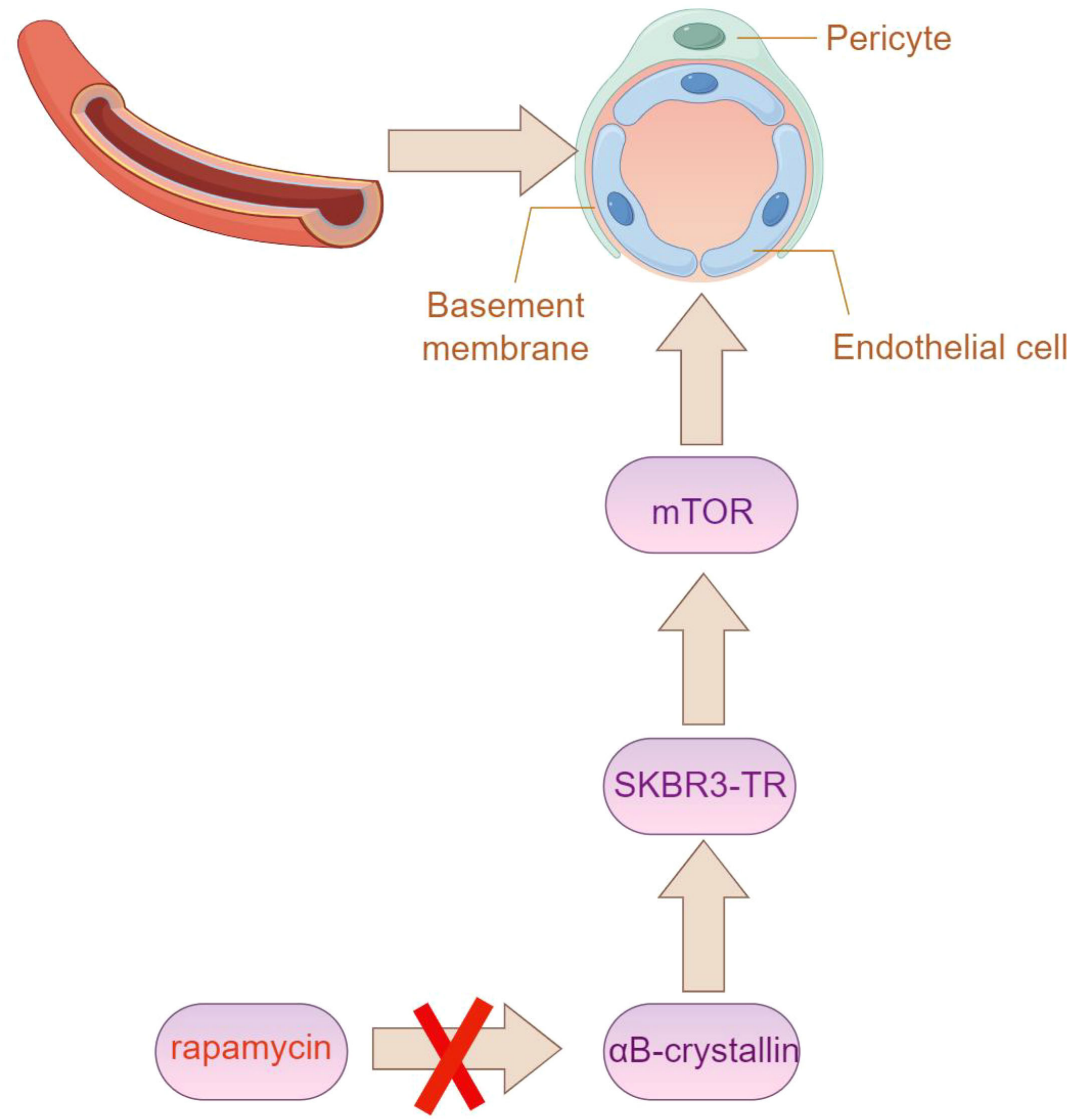
The expression of Alpha-B-crystallin (aB-crystallin) is elevated in SKBR3-TR cells. Silencing this protein notably impairs tube formation induced by SKBR3-TR cells, along with the activation of the mechanistic target of rapamycin (mTOR) in endothelial cells. (By Figdraw).

The relationship between trastuzumab resistance and angiogenesis remains unclear; however, multiple studies suggest that, in contrast to trastuzumab-sensitive SKBR3 cells, the culture supernatant from trastuzumab-resistant SKBR3 cells (referred to as SKBR3-TR) significantly enhances endothelial cell elongation, providing valuable insights. Alpha-B-crystallin, found in vascular endothelial cells, plays an important role in tumor angiogenesis by regulating vascular structure (54). Interestingly, Alpha-B-crystallin (aB-crystallin) is highly upregulated in SKBR3-TR cells. Silencing aB-crystallin significantly reduces tube formation induced by SKBR3-TR and simultaneously inhibits the activation of the mechanistic target of rapamycin (mTOR) in endothelial cells (**Figure 6**). Moreover, rapamycin, an mTOR inhibitor, successfully reverses the tube formation promoted by SKBR3-TR. Overall, aB-crystallin enhances the ability of SKBR3-TR cells to activate mTOR in endothelial cells, thus facilitating angiogenesis (55).

The mTOR inhibitor rapamycin affects the formation of tube-like structures induced by SKBR3-TR cells51 (56). Research shows that treating human aortic endothelial cells (HAECs) with SKBR3-TR cell culture medium increases the amount of phosphorylated mTOR (p-mTOR) in HAECs. However, adding rapamycin to HAECs co-cultured with SKBR3-TR cells reduces p-mTOR levels.Additionally, HAECs stimulated with culture medium treated with SKBR3-TR cells form tube-like structures, and adding rapamycin further inhibits this formation.The results indicate that tube-like structure formation induced by SKBR3-TR cells is related to mTOR activation, and the mTOR inhibitor rapamycin can reverse the promotion of tube-like structure formation induced by SKBR3-TR cells, offering potential in addressing trastuzumab resistance (12, 57).

## 9 Tumor-associated regulatory factors

### 9.1 Transcription factors, redox environment

Unlike typical RNA molecular structures, circular RNA (circRNA) lacks 3’ or 5’ ends, and its unique spatial configuration is closely associated with the specific progression of diseases.Studies have shown a strong correlation between circRNA and resistance in breast cancer (58). circCDYL2 promotes trastuzumab resistance by maintaining HER2 downstream signal transduction.Specifically, circCDYL2 stabilizes GRB7 forming a circCDYL2-GRB7-FAK complex, and maintaining the activity of downstream signaling molecules AKT and ERK1/2.Studies have shown that HER2+ breast cancer patients with high expression of circCDYL2 have poorer outcomes and shorter disease-free survival (DFS) and overall survival (OS) after trastuzumab treatment. Therefore, to some extent, circRNA could be a potential research direction for reducing trastuzumab resistance (59, 60).

### 9.2 Autophagy evasion

Autophagy is a multistep process that allows cells to maintain homeostasis under stress by degrading dysfunctional or unwanted intracellular components. Growing evidence suggests that autophagy plays a crucial role in tumorigenesis, including processes such as tumor formation, growth, invasion, metastasis, dormancy, and resistance to therapy. In a three-dimensional *in vitro* model, chronic trastuzumab exposure was found to increase autophagy and sustain cell survival, leading to trastuzumab resistance (58). In breast cancer, autophagy has emerged as a novel resistance mechanism for anti-HER2 therapies. Studies suggest that autophagy not only aids in cellular stress responses but may also help tumor cells evade therapeutic effects by promoting survival. For instance, trastuzumab-resistant cells displayed increased autophagic activity, which correlated with their ability to resist the drug. Therefore, targeting autophagy may present new opportunities for overcoming trastuzumab resistance.

Targeting autophagy-related protein 5 (ATG5), a protein involved in autophagosome precursor formation, has been shown to suppress autophagy and reverse trastuzumab resistance in HER2-positive breast cancer. ATG5 plays a key role in autophagy, and loss of its function could reduce cellular tolerance to chemotherapeutic agents. Although trastuzumab is initially effective in treating HER2-positive breast cancer, resistance eventually develops in many patients (51, 59). By targeting ATG5, autophagy can be effectively inhibited, thus enhancing trastuzumab’s efficacy and overcoming drug resistance. This finding highlights the importance of autophagy in drug resistance mechanisms and offers new insights for treating HER2-positive breast cancer.

Autophagy inhibitors and bioactive molecules can enhance the efficacy of breast cancer treatments. Autophagy inhibitors prevent cancer cells from evading therapy by blocking autophagy. Chloroquine, a commonly used autophagy inhibitor, can inhibit autophagy and sensitize breast cancer cells to chemotherapy. Other inhibitors, such as hydroxychloroquine and bafilomycin A1 (BafA1), also suppress autophagy. Bioactive molecules, including inhibitors of AMPK (AMP-activated protein kinase) and mTOR (mechanistic target of rapamycin), can interfere with autophagy-regulating pathways in breast cancer cells, thereby enhancing chemotherapy effectiveness (57, 60).

In addition, some miRNAs and lncRNAs have been found to regulate autophagy in breast cancer cells. By modulating the expression of these molecules, the efficacy of anti-cancer treatments can be improved (60). In summary, using autophagy inhibitors and bioactive molecules to interfere with the autophagy process in breast cancer cells can improve the anti-cancer effects of drugs. This provides new insights into developing more effective strategies for treating breast cancer (61).

## 10 Impaired lysosomal function

Antibody-drug conjugates (ADCs) are internalized by cells through binding to specific target molecules, followed by receptor-mediated endocytosis, ultimately reaching the lysosome (62). Within the acidic environment of lysosomes, ADCs undergo chemical or enzymatic cleavage, releasing cytotoxic drugs that then exert their lethal effects on cancer cells. However, the efficacy of ADCs can be compromised due to various factors, leading to reduced effectiveness. One key mechanism of resistance involves the transport of cytotoxic drugs from the lysosomal lumen to the cytosol. This mechanism is particularly relevant for ADCs with non-cleavable linkers, which may depend on specialized transporters to facilitate drug transfer from lysosomes to the cytosol. Abnormalities in the expression levels or function of these transporters can impede drug release, thereby contributing to increased ADC resistance (63).

Studies have investigated the mechanism of T-DM1 resistance by isolating three distinct resistant HER2-positive clones. Compared to the parental clones, these resistant clones maintained similar HER2 expression levels, with no changes in internalization and trafficking pathways. However, one clone showed elevated lysosomal pH levels and disrupted proteolytic activity, leading to the accumulation of T-DM1. This lysosomal dysfunction impaired T-DM1 processing, ultimately reducing its antitumor efficacy. Understanding this functional defect not only sheds light on the various mechanisms of ADC resistance but also provides potential strategies for overcoming such resistance (16).

## 11 Cell migration

The formation of heterodimers results in the recruitment of downstream signaling molecules, which inevitably influences cell movement through this recruitment process. Cell movement, a complex and multi-step process, involves actin remodeling, focal adhesion (FA) turnover, and other regulatory factors, requiring dynamic regulation of FA assembly and disassembly (43). Proteins such as c-Src and focal adhesion kinase (FAK) play key roles in regulating FA formation and turnover. Studies have shown that Heregulin (HRG) triggers a precise sequence of events via the HER2/HER3 signaling pathway, altering cell morphology and promoting migration in HER2-positive breast cancer cells. Specifically, HRG induces actin cytoskeleton reorganization and FA complex formation in BT-474 breast cancer cells, facilitating actin nucleation.

This signaling cascade is activated by HER2/HER3, leading to the involvement of kinases and scaffolding proteins like c-Src, FAK, and paxillin. Phosphorylation of paxillin recruits PAK1 kinase, which further phosphorylates cortactin. Simultaneously, paxillin interacts with N-WASP, co-regulating the Arp2/3 complex, resulting in local actin reorganization. These changes ultimately enhance cell migration. HRG enhances cell migration by modifying cell morphology and rearranging the actin cytoskeleton in HER2-positive breast cancer cells, involving numerous kinases and scaffolding proteins, including c-Src, FAK, paxillin, PAK1, cortactin, and N-WASP. These findings are crucial in understanding the molecular mechanisms underlying migration in HER2-positive breast cancer cells and drug resistance, providing a foundation for developing novel and more specific therapeutic strategies to disrupt the progression and metastasis of HER2-positive breast cancer (46).

## 12 Discussion

Breast cancer that overexpresses Human Epidermal Growth Factor Receptor 2 (HER2+) due to gene amplification or overexpression constitutes 15-20% of all breast cancer cases. The specific mechanisms of resistance to trastuzumab are currently unclear. This paper aims to review the existing research on the resistance mechanisms of trastuzumab, based on its target, from aspects such as genetic loci, molecular structure, signaling pathways, and the tumor microenvironment and to outline current research progress and new strategies.

The activation of HER family members primarily depends on autocrine or paracrine pathways. ErbB receptors possess an extracellular segment of approximately 630 amino acids, organized into four distinct structural domains: I/L1, II/CR1, III/L2, and IV/CR2. These domains are arranged in tandem, alternating with double-domain units. Additionally, the receptors contain a single transmembrane region and a cytoplasmic tyrosine kinase. Each structural domain includes specific sub-domains and amino acid residues, which can be classified into ligand-binding and dimerization domains. Except for HER3, all receptors contain a cytoplasmic tyrosine kinase region. Except for HER2, all receptors bind specific ligands through their extracellular domains. Upon dimerization, HER2 exhibits strong kinase activity and preferential binding. The dimer features leucine-rich residues in extracellular sub-domains I and III and cysteine residues in sub-domains II and IV, primarily connected by disulfide bonds.

Heterodimerization triggers the phosphorylation of tyrosine kinases, subsequently leading to the activation of several signaling pathways, including Ras/MAPK, PI3K/AKT/mTOR, PLCγ/PKC, and JAK2/STAT. These pathways regulate essential cellular processes, such as proliferation, apoptosis, differentiation, angiogenesis, and invasiveness.

Following anti-HER2 treatment, gene overexpression or deletion can result in mutations within the target itself, causing reduced or absent HER2 expression, which diminishes drug sensitivity. Studies on trastuzumab resistance have identified mutations in genes such as ATM, CDH1, GNAS, MLH1, RB1, SMARCB1, SMO, TP53, and VHL. Notably, TP53 mutations are the most frequently observed, particularly in circulating cell-free DNA (cfDNA) from patients with HER2-positive metastatic breast cancer. Additionally, breast cancer is the most common malignancy (25.5%) among women harboring pathogenic TP53 mutations. Importantly, the p53 signaling pathway plays a crucial role in the cellular response to DNA damage, highlighting its significance in the stress response. These genetic alterations can contribute to therapeutic resistance.

Given the widespread occurrence of mutations, splicing, and post-translational modifications in tumor cells, HER2 inevitably produces isoforms and various subtypes. Current research has shown the presence of HER2 splicing variants that lack exon 16 (HER2Δ16), and the co-expression of HER2 subtypes exerts a unique influence on breast tumor phenotypes. Oncogene activation, which is predominantly linked to epithelial-mesenchymal transition (EMT), is integral to this process. The formation of stable disulfide bonds at the molecular level ultimately activates downstream pathways, potentially leading to delayed responses and decreased efficacy of trastuzumab.

Moreover, variability in tumor antigen expression can influence the effectiveness of trastuzumab-based drug conjugates. Trastuzumab deruxtecan (T-DXd), an antibody-drug conjugate (ADC), was evaluated in the KRISTINE trial, a phase II study involving T-DM1 combined with pertuzumab in neoadjuvant therapy. HER2 heterogeneity was defined as HER2-negative regions detected by fluorescence *in situ* hybridization (FISH) in 10% of cases, or ERBB2 gene amplification detected in more than 5% but fewer than 50% of tumor cells. None of the ten patients exhibiting HER2 heterogeneity reached the primary endpoint of a pathologic complete response (pCR) of 0%. The study results indicated that patients with higher HER2 heterogeneity had poorer progression-free survival (PFS) and overall survival (OS) compared to those with lower heterogeneity.

Under specific conditions, proteolytic cleavage may give rise to certain mutant forms of the epidermal growth factor receptor. For instance, soluble EGFR (sEGFR) can be generated either by selective mRNA splicing of the receptor or by site-specific proteolytic cleavage, which releases the extracellular domain. Similarly, cleavage of HER2-ECD by matrix metalloproteinases (MMPs) or ADAM (A Disintegrin And Metalloprotease domain) produces a truncated version of HER2, known as p95HER2—a 95 kDa truncated form of the HER2/neu protein. The absence of the extracellular domain in p95HER2 results in trastuzumab resistance, as p95HER2 forms homodimers that bypass trastuzumab binding or reduce its efficacy, thus activating various downstream signaling pathways. This variant is found in approximately 22.4% of cancer patients and is closely associated with breast cancer metastasis and recurrence.

Additionally, the truncated version of the antigen’s extracellular domain could represent another resistance mechanism for trastuzumab deruxtecan that has yet to be clinically demonstrated. This phenomenon has previously been observed with trastuzumab: tumors expressing the full-length receptor respond well, whereas those expressing truncated p95HER2 are resistant.

The resistance rate to trastuzumab in clinical settings is increasingly significant. Although the mechanisms of resistance are not fully understood, they can be investigated through genetic loci, molecular structure, signaling pathways, and the tumor microenvironment to develop targeted interventions.

As the primary target of trastuzumab, anti-HER2 treatment often leads to overexpression or absence of related genes, primarily manifested as mutations in ATM, CDH1, GNAS, MLH1, RB1, SMARCB1, SMO, TP53, and VHL. Among these, TP53 mutations are the most common (25.5%), with major variants being p.Ser241Phe and c.376-2dup. Combining HER2-targeted drugs with anti-mutp53 therapy may synergize in treating HER2-positive breast cancer patients. Moreover, mutations such as those in ATM and RB1 cannot be disregarded. Targeting trastuzumab resistance using small molecule tyrosine kinase inhibitors (TKIs) alone or in combination with monoclonal antibodies or ADCs has shown some effectiveness; however, the mechanisms of resistance due to gene overexpression remain unclear and require further investigation.

HER2 expression is influenced by its mutations, density, and isoforms. The main variant, currently HER2Δ16, lacks exon 16, resulting in crosstalk through epithelial-mesenchymal transition and the formation of intermolecular disulfide bonds, which delays response and reduces trastuzumab efficacy. Proteolytic cleavage of HER2-ECD, resulting in the truncated form p95HER2, and the formation of intermolecular homodimers can also contribute to trastuzumab resistance.

HER2 lacks specific antibodies and forms dimers with other ligands through amino acid residues, exhibiting strong kinase activity and binding preference. The exposure sufficiency of HER2 dimers after their formation affects trastuzumab target efficiency. Antibody binding impediment is a primary mechanism of trastuzumab resistance, mainly involving mucin 4 (MUC4) and CD44. Multi-channel targeted delivery, such as KLA-R16, survivin siRNA, and trastuzumab, can alleviate recurrent breast cancer resistance caused by spatial barriers. Additionally, trastuzumab-derived HER2-specific CAR T cells specifically target resistant cells with spatial barriers, showing promising prospects for further research. Elevated levels of IGF2 protein can lead to the formation of IGF1R/HER2 heterodimers. Moreover, AXL, through binding with its ligand growth arrest-specific protein 6 (GAS6) and extracellular domain-mediated dimerization, or by forming signal cascades with other transmembrane receptors to activate PI3K/AKT and MAPK pathways, can also contribute to trastuzumab resistance. Drug conjugation strategies, such as targeting AXL alongside anti-HER2 drugs, may represent a potential treatment approach for trastuzumab resistance.

Activation of the downstream signaling pathways of HER2 is a key issue in trastuzumab resistance, involving constitutive activation or crosstalk of signaling pathways, and various regulatory factors such as the loss of phosphatase and tensin homolog (PTEN), leading to constitutive activation of the PI3K/AKT pathway. Various small molecular proteins that induce changes in cell conformation also affect key downstream signaling pathways, such as the overactivation of the HER2-SHCBP1-PLK1 axis by SHC linker protein 1 (SHC1), representing a novel mechanism of cancer resistance to trastuzumab. Activation of the cyclin D1-CDK4/6 (cyclin-dependent kinase 4/6) axis also plays a significant role in trastuzumab resistance, and CDK4/6 inhibitors combined with anti-HER2 and endocrine therapy have demonstrated certain therapeutic benefits.

At the submicroscopic level, the tumor microenvironment plays a significant role in the interactions between receptors and small molecular proteins, primarily involving regulation, immune escape, and functional impairment of antibody-dependent cellular cytotoxicity (ADCC), with multiple immune cells influencing cytotoxic responses that are closely linked to resistance. Trastuzumab-resistant tumor cells exhibit high proliferation of endothelial cells, with alpha-B-crystallin in vascular endothelial cells regulating vascular morphology. Additionally, alpha-B-crystallin enhances the ability of SKBR3-TR cells to activate mTOR in endothelial cells, promoting angiogenesis and contributing to trastuzumab resistance. Experiments have demonstrated that mTOR inhibitors can inhibit this process, offering a potential solution to trastuzumab resistance.

Tumor-associated regulatory factors exert regulatory effects on downstream HER2 signaling, with possible mechanisms involving competitive inhibition, prevention of degradation of key downstream proteins, and inhibition of ubiquitin-mediated degradation. For instance, the truncated peptide segment (β-TrCP-343aa) encoded by circ-β-TrCP competitively binds with NRF2, preventing NRF2 protein degradation mediated by SCF β-TrCP, and the protective effect of β-TrCP-343aa on NRF2 protein requires GSK3 activity. Subsequently, elevated transcription of NRF2 upregulates a series of antioxidant genes, leading to trastuzumab resistance, and provides a potential therapeutic target to overcome trastuzumab resistance. Additionally, evasion of autophagy and cell migration are emerging areas in the study of trastuzumab resistance, involving signal transduction of various kinases and scaffolding proteins, providing a broad research field and potential for developing novel, more specific treatment strategies to intervene in the progression and metastasis of HER2-positive breast cancer.

Moreover, variability in tumor antigen expression can influence the effectiveness of trastuzumab-based drug conjugates. Trastuzumab deruxtecan (T-DXd) is an antibody-drug conjugate (ADC) consisting of a humanized anti-HER2 monoclonal antibody covalently linked to the topoisomerase I inhibitor DXd. The high drug-to-antibody ratio (8:1) ensures that, after T-DXd internalizes and its adaptors are cleaved, high concentrations of DXd are delivered to the target tumor cells. The membrane permeability of DXd allows it to diffuse through the cell membrane, exerting antitumor activity against surrounding tumor cells, regardless of HER2 expression. The ADC structure is complex, and resistance can develop at various stages, including antigen expression, recognition, internalization, degradation, and drug release. Antigen-associated resistance mechanisms include reduced HER2/NECTIN4 levels, tumor heterogeneity, and truncation of the antigen extracellular domain. Studies suggest that reduced HER2 levels may lead to refractory disease, while tumor heterogeneity affects ADC efficacy, leading to worse progression-free survival (PFS) and overall survival (OS). The KRISTINE and ZEPHIR trials demonstrated that patients with higher tumor heterogeneity had poor treatment outcomes. Antigen truncation, such as p95HER2, can also bypass trastuzumab binding, leading to drug resistance.

In addition to antigen resistance, tumor cells may also develop resistance to the drug payload. Upregulation of drug efflux transporters ABCC2 and ABCG2 has been found to reduce intracellular drug accumulation, resulting in resistance. Furthermore, binding chemistry, drug-to-antibody ratio (DAR), and binding sites affect ADC efficacy, and optimizing DAR and binding methods is crucial to balancing efficacy and tolerance.

Lysosomal dysfunction and activation of signaling pathways are also mechanisms of drug resistance. Impaired lysosomal acidification can limit drug release, while upregulation of the PI3K/AKT/mTOR and Wnt/β-catenin pathways is also associated with resistance.

The mechanisms of resistance to trastuzumab require further exploration to develop effective solutions.
